# Environment predicts *Batrachochytrium dendrobatidis* lineage distribution and zones of recombination in South Africa

**DOI:** 10.1002/ece3.11037

**Published:** 2024-02-22

**Authors:** Ruhan Verster, Pria N. Ghosh, Tom R. Sewell, Trenton W. J. Garner, Matthew C. Fisher, Wynand Muller, Dirk Cilliers, Ché Weldon

**Affiliations:** ^1^ Unit for Environmental Sciences and Management North‐West University Potchefstroom South Africa; ^2^ MRC Centre for Global Infectious Disease Analysis, School of Public Health Imperial College London London UK; ^3^ Institute of Zoology Zoological Society of London London UK

**Keywords:** Africa, amphibian chytrid fungus, distribution modeling, environmental envelope, predictive ecological niche modeling, recombinant zone

## Abstract

The amphibian‐infecting chytrid fungus, *Batrachochytrium dendrobatidis* (*Bd*), is widespread throughout Africa and is linked to declines of populations and species across the continent. While it is well established that the lineage of *Bd* encodes traits which determine disease severity, knowledge around how lineages are distributed according to environmental envelope is unclear. We here studied the distribution of *Bd* in South Africa based on the two lineages found, *Bd*GPL and *Bd*CAPE, in terms of their genome and environmental envelope statistically associated with their distribution. We used *Bd* surveillance data from published studies, as well as data collected during fieldwork from across South Africa, Lesotho, and eSwatini with samples collected along a transect spanning most of South Africa from Lesotho to the west coast. We utilized lineage‐typing qPCR to resolve the spatial distribution of *Bd*GPL and *Bd*CAPE across South Africa and used the resulting surveillance data to create a predictive ecological niche model for *Bd* lineages in South Africa. Phylogenomic analyses were performed on isolates sourced from across the transect. We show that *Bd*GPL demonstrates a strong isolation by distance suggestive of stepping‐stone dispersal, while *Bd*CAPE showed two distinct clusters within their genomic structure that appear geographically and temporally clustered, indicating two separate invasions. Our predictive niche model revealed that the two lineages tended to occur in different ecotypes; *Bd*GPL was associated with lower altitude, arid regions while *Bd*CAPE occurred across cooler, higher altitude environs. Niche predictions identified a zone of lineage contact, where genomics identified inter‐lineage recombinants. We argue that this zone of recombination should be prioritized for disease surveillance as it is a potential hotspot for the evolution of variants of amphibian chytrid with novel traits that may be epidemiologically relevant.

## Graphical abstract

The amphibian chytrid fungus has been seen to be widespread around the globe and consist of different genetic lineages. This fungus has recently been found to be able to form recombinant lineages between these existing genetic lineages, with still unknown consequences for the amphibian host. In South Africa, it was found that the hypervirulent global lineage has a wide environmental envelope creating the possibility for a number of contact zones where interlineage genetic recombination can occur, leading to recombinant lineages and potential new threats to the amphibian hosts.

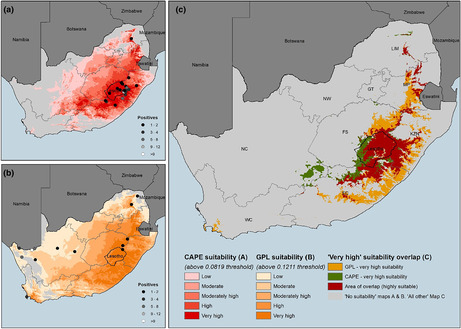

## 1 INTRODUCTION

Across our globalized planet, pathogens are frequently translocated, sparking epidemics that threaten human, livestock, crop, and wildlife health. Pathogens, once established, go on to adapt to naïve hosts through the processes of mutation, lineage sorting, and recombination—processes that can lead to a changing epidemiological landscape. Global‐scale pandemics are characterized by successive waves of emergence as genetically distinct lineages spread. However, while better understood for viruses such as SARS‐CoV‐2 (Harvey et al., 2021), we have poor understanding of the extent that the epidemiology of fungal pathogens owes to lineage‐specific traits that characterize emerging lineages. We have even less understanding of the evolutionary changes that occur in pathogens when recombination generates novel genotypes. These evolutionary “jumps” have the potential to lead to phenotypic changes of large consequence leading to lineage replacement by newer, fitter variants that may propagate spatiotemporal waves of infection (Fisher et al., 2012)

*Batrachochytrium dendrobatidis*, (*Bd*), and *B. salamandrivorans* are two pathogenic chytrid fungi that have emerged to infect amphibians around the globe (Martel et al., 2013). The disease that they cause, chytridiomycosis, has contributed to the decline of over 500 amphibian species and is believed to be responsible for the extinction of over 90 species (Scheele et al., 2019). The epidemiology of chytridiomycosis is complicated by the fact that *Bd* to date is known to comprise of at least five lineages, with observed differences in infectivity and virulence when tested (Byrne et al., 2019; Farrer et al., 2011; Fisher et al., 2021; Fu & Waldman, 2019; Ghosh et al., 2021; O'Hanlon et al., 2018). The geographical distributions of these lineages vary globally, and inter‐lineage contact zones appear to generate genetic mosaics, demonstrating the existence of inter‐lineage recombination (Byrne et al., 2019; Jenkinson et al., 2016; O'Hanlon et al., 2018).

Amphibians in South Africa are known to be parasitized by two lineages of *Bd—*the globally widespread *Bd*GPL and rarer *Bd*CAPE which is largely found in Western and Southern Africa (Doherty‐Bone et al., 2020; Ghosh & Fisher, 2016). The African continent is unusual with respect to *Bd* genomic diversity, as both lineages are widespread, and it is the globally rarer lineage, *Bd*CAPE, that has been shown to be the cause of amphibian mortality and population declines (Weldon et al., 2020). Environmental niche modeling has correlated *Bd* distribution in South Africa with cooler, wetter areas including where these conditions prevail for river catchments in predominantly dry regions (Tarrant et al., 2013). Disease surveillance within these regions of high environmental suitability for occurrence have subsequently revealed an overlapping distribution of these two lineages (Ghosh et al., 2021). Of conservation concern is the tendency of recombinant fungal pathogens to be more virulent than either of the parent lineages, to result in expanded host ranges (Brasier, 2000; Jenkinson et al., 2016; Walker et al., 2011), or to widen their geographical niche through the generation of diverse traits.

Here, we describe a multi‐year broad‐scale molecular genetic surveillance of *Bd* utilizing lineage‐specific markers and genomic epidemiology. We focus on the Orange River system which spans a wide environmental gradient across altitude, temperature, and rainfall, and is commonly inhabited by frogs of the genus *Amietia*, all of which are known to be readily infected with *Bd* (Griffiths et al., 2018; Smith & Weldon, 2007; Weldon & Du Preez, 2006). Our analyses identify a suite of abiotic environmental factors that are associated with the distribution of *Bd* lineages and identifies a zone of lineage co‐occurrence and inter‐lineage recombination. This study therefore presents an opportune use of pathogen landscape genetics and ecological niche modeling to understand emergent patterns of pathogen distributions that can lead to disease.

## 2 MATERIALS AND METHODS

### 2.1 Field sampling

The Orange River was selected as a natural transect for this survey as it runs approximately 2300 km from the origin in the Lesotho highlands to the mouth at Alexander Bay where it enters the Atlantic Ocean (Lange et al., 2007). The river crosses cooler, summer rainfall regions at the origin, to the arid and warmer region along the western coast of South Africa (Cambray et al., 1986; Conley & Van Niekerk, 2000).

Field sites were focused on wetlands and other water bodies along the Orange River basin. There were 32 sites in total located across 1500 km from Vioolsdrift, at 155 m elevation at the most western site of the transect, to Lotheni in the Ukhamlamba Drakensberg National Park, at 1600 m elevation and the most eastern site of the transect. Areas where sites allowed for multiple sampling option within a 10 km radius were treated as single sites. At these sites, tadpoles and adult amphibians were collected by using dipnets or catching them by hand, respectively.

DNA was collected from the ventral surface of adult amphibians using sterile swabs, as well as from toe‐clips from adult frogs for *Bd* isolation (Fisher et al., 2018). Tadpoles were euthanized by submersion in MS‐222 solution (0.2%) before mouthparts were excised and preserved in 70% ethanol or used for *Bd* isolation (Fisher et al., 2018). Because the range of the study area did not allow for a single species to be selected, sampling mostly focused on, but was not restricted to, members of the genus *Amietia* since it has representative species within the entire study area (Figure S1).

### 2.2 Lineage identification of swab samples

Before lineage‐specific qPCRs were run on field swab samples, we ran a pan‐lineage ITS‐PCR to determine if samples were positive for *Bd* (Boyle et al., 2004). Samples that amplified in duplicate with a GE > 0.1 were then subjected to lineage‐specific qPCR, with primers designed for *Bd*GPL and *Bd*CAPE, using methods developed by Ghosh et al. (2021). All samples were run in duplicate, with two negative controls (for which dH_2_O replaced the sample), and quantitation standards, created from zoospores harvested from confirmed *Bd*GPL and *Bd*CAPE isolates (Ghosh et al., 2021; O'Hanlon et al., 2018).

### 2.3 Genomic analysis of isolates

Isolates were whole genome sequenced (WGS) as part of the work published by O'Hanlon et al. (2018). Briefly, these isolates were extracted and purified DNA was quality assured prior to library preparation with the Illumina HiSeq™ high output V4 chemistry and sequenced on an Illumina HiSeq™ machine (125 + 125 bp paired‐end). Sequenced reads were aligned back to the *Bd*JEL423 reference genome using BWA‐mem (Li & Durbin, 2009) and variant detection was performed using freebayes version dbb6160 (Garrison & Marth, 2012). High‐quality variants were filtered using vcftools (Li et al., 2009) by applying the filtration steps outlined in O'Hanlon et al. (2018). We then generated a multi‐sample VCF of all South African isolates using vcftools (Danecek et al., 2011). The combined VCF file was used to generate a Minimum Spanning Network (MSN; showing the genetic distance, Nei, between isolates) and a Principal Coordinate Analysis (showing separation of lineages) and isolation by distance analyses for each lineage.

We created the MSN using the poppr package (v2.8.1); the Principal Coordinate Analysis was generated using the adegant package (v2.1.1) and visualized using ggplot2 (v3.1.0). The isolation by distance analyses shows the correlation between geographic (Euclidian) distance and genetic distance (Nei) for each lineage. Euclidian distances were calculated using the R function dist in the R stats package (v3.5.1), and the genetic distance Nei, which assumes that genetic distance arises due to mutation and genetic drift, was calculated using the StAMPP package (v1.5.1). A Mantel test was carried out to assess if correlations between the genetic and geographic distances were statistically significant. An isolation by distance (IBD) model assumes genetic differentiation as a function of distance with a high *R* ^2^ value indicating that genetic distance and geographic distance are correlated. IBD does not consider any geographic complexity that may predict population structure and is used here as exploratory baseline analysis to investigate whether either lineage deviates from a simple genetic distance/geographical distance correlation.

### 2.4 Site climate characterization

We characterized all *Bd* positive sites in terms of mean temperature (average daily surface temperature between 1981 and 2018), mean daily precipitation (per day averaged from 1981 to 2018), and mean annual dew/frost point at 2 m above ground level (daily average between 1981 and 2018). Mean temperature, mean daily precipitation, and mean annual dew point (https://power.larc.nasa.gov) were selected based on previous work by Puschendorf et al. (2009), Tarrant et al. (2013), and Murray et al. (2011). The MERRA‐2 database was used to obtain data for these parameters with a resolution of 0.5° × 0.625° lat/lon calculating to approximately 50 km latitudinal distance. Kruskal‐Wallis tests were used to determine if significant differences could be found between the medians of the environmental parameter groups, elevation, as well as prevalence at sites with *Bd*, due to data having a non‐normal distribution. These tests were done with the environmental parameters per site in order to determine if sites were significantly different where the different lineages were found. Elevation was predicted to correlate with other variables, so we used a Pairwise Wilcox test on significantly different variables to identify groups that differed from each other, using Holm as the *p*‐value adjustment method. The Merra‐2 dataset was used due to its public availability, which allowed for the initial characterization of the general environmental envelope per lineage.

### 2.5 Predictive distribution modeling

Initially the Merra‐2 database was used to do site characterization of the *Bd* lineages found. This allowed for the rough characterization of areas suitable with the needs of the different lineages. After sampling along a transect spanning both lineages' climatic envelope, the results were used to construct lineage distribution models, using the finer scale information available from WorldClim (https://www.worldclim.org/data/bioclim.html).

We modeled lineage distributions using MaxEnt (version 3.4.4; Phillips et al., 2006) described by Elith et al. (2011) to correlate environmental parameters with the presence of either *Bd* lineage. A database was constructed from the records of *Bd* sampling in South Africa (accessible on the DRYAD database), but for the models, only positive sites were used with duplicate coordinates removed. Several studies have demonstrated the effectiveness of MaxEnt as a distribution modeling approach (Gibson et al., 2007; Hernandez et al., 2008; Pearson et al., 2007; Phillips et al., 2006; Stabach et al., 2009; Ward, 2007). In comparative analyses, MaxEnt has been shown to either outperform or perform comparably to models such as BIOCLIM, generalized linear models, random forest, support‐vector machine, and DOMAIN (Yudaputra et al., 2019), as well as to ensemble approaches combining random forest, support‐vector machine, MaxLike, boosted regression trees, classification and regression trees, flexible discriminant analysis, and generalized linear models (Kaky et al., 2020).

A database for all known *Bd* records for South Africa, Lesotho, and Eswatini was created with the following information: locality and coordinates of sample origin, sample size, prevalence of *Bd*, lineage found (if available), date of sampling, and host genus and species name, with multiple samples seen as a single entry. Bioclimatic variables (30 s resolution) as well as the SRTM elevation dataset (30 s resolution) used for modeling were obtained from WorldClim (https://www.worldclim.org/data/bioclim.html). Prior to modeling, these variables were tested for inter‐correlations, and only those exhibiting a correlation coefficient below the threshold of 0.7 were included in the analysis to ensure model robustness.

The use of the MaxEnt for predicting species' geographical distribution often involves default parameters, which could lead to complex and potentially overfitted models that are challenging to interpret, as highlighted by Radosavljevic and Anderson (2014) and Warren et al. (2014). To address this, Muscarella et al. (2014) introduced the R package ENMeval, which optimizes MaxEnt model parameters by assessing various combinations of regularization multipliers and features through metrics like AUC, AICc, and omission rates to mitigate overfitting. This approach facilitates the selection of model parameters that yield less complex models, thereby enhancing the reliability of the results and avoiding the biases that may arise from unvalidated user‐selected parameters.

We used the ENMeval package within R version 4.0.2 to test for model parameters. This package was employed to assess an array of regularization multipliers (RM) alongside five feature types—linear (L), quadratic (Q), hinge (H), product (P), and threshold (T)—as per Phillips et al. (2006), with the model's fit and complexity judged by the corrected Akaike information criterion (AICc), while the AUC difference and 10% training omission rate gauged the potential overfitting (Akaike, 1973; Burnham & Anderson, 2004). The optimal parameter set for constructing each model was identified by the lowest delta.AICc value. For both the *Bd*GPL and *Bd*CAPE models, this meant an RM of 0.5 with features LQ (Tables S2 and S3).

The two models were subsequently executed using MaxEnt, wherein for each, a random subset constituting 80% of the positive instances was employed for training purposes, and the remaining 20% was utilized to validate the precision of the model's predictive capabilities. Model iterations were configured to 100 replications using the subsample method, with the random seed option activated to ensure reproducibility, while all other parameters were retained at their default values.

Model performances were evaluated using the area under curve (AUC) of the receiving operating characteristic (ROC) curve and by using jack‐knife tests. The jack‐knife tests examined the importance of each variable, firstly by removing one variable at a time and secondly by testing each variable in isolation (Figures S1 and S2). AUC values of 1 indicates perfect models, while values of 0.5 indicates that the model has no predictive ability. The “10 percentile training presence logistic threshold” was used to distinguish between suitable and non‐suitable areas (*Bd*GPL = 0.1211; *Bd*CAPE = 0.0819), while the suitable areas were classified in to five suitability classes using a Natural Breaks (Jenks) method. The highest class of suitability for *Bd*GPL and *Bd*CAPE were then overlaid and further assessed.

## 3 RESULTS

### 3.1 *Bd* lineage abundance and distribution

The lineage of 204 samples (from the total positive samples of 270 that included 59 samples that were collected before 2015 fieldwork) were determined through lineage‐specific qPCR of which approximately 72% of samples belonged to the *Amietia* genus. The most abundant species was *Amietia delalandii* (*n* = 99), while other species belonging to this genus accounted for the remaining 54 individuals (full species list in Table S1). In addition, 40 *Bd* isolates were cultured and subjected to whole genome sequencing (O'Hanlon et al., 2018). Collected isolates that were whole genome sequenced were either *Bd*CAPE, *Bd*GPL, or a recombinant lineage. This recombinant was identified using isolate clustering through principal component analyses using a subset of 3900 SNPs (O'Hanlon et al., 2018). In total, 17.78% of positives samples were identified to be *Bd*GPL, 47.41% determined to be *Bd*CAPE, 10.37% tested positive for both lineages, and 24.44% of positives could not be defined to lineage level. These unspecified positive samples were all part of the historical sample matrix and were collected prior to 2002, at which time isolation of cultures were not a focus for the sampling strategies, and lineage specific primers were not yet available.

**FIGURE 1:**
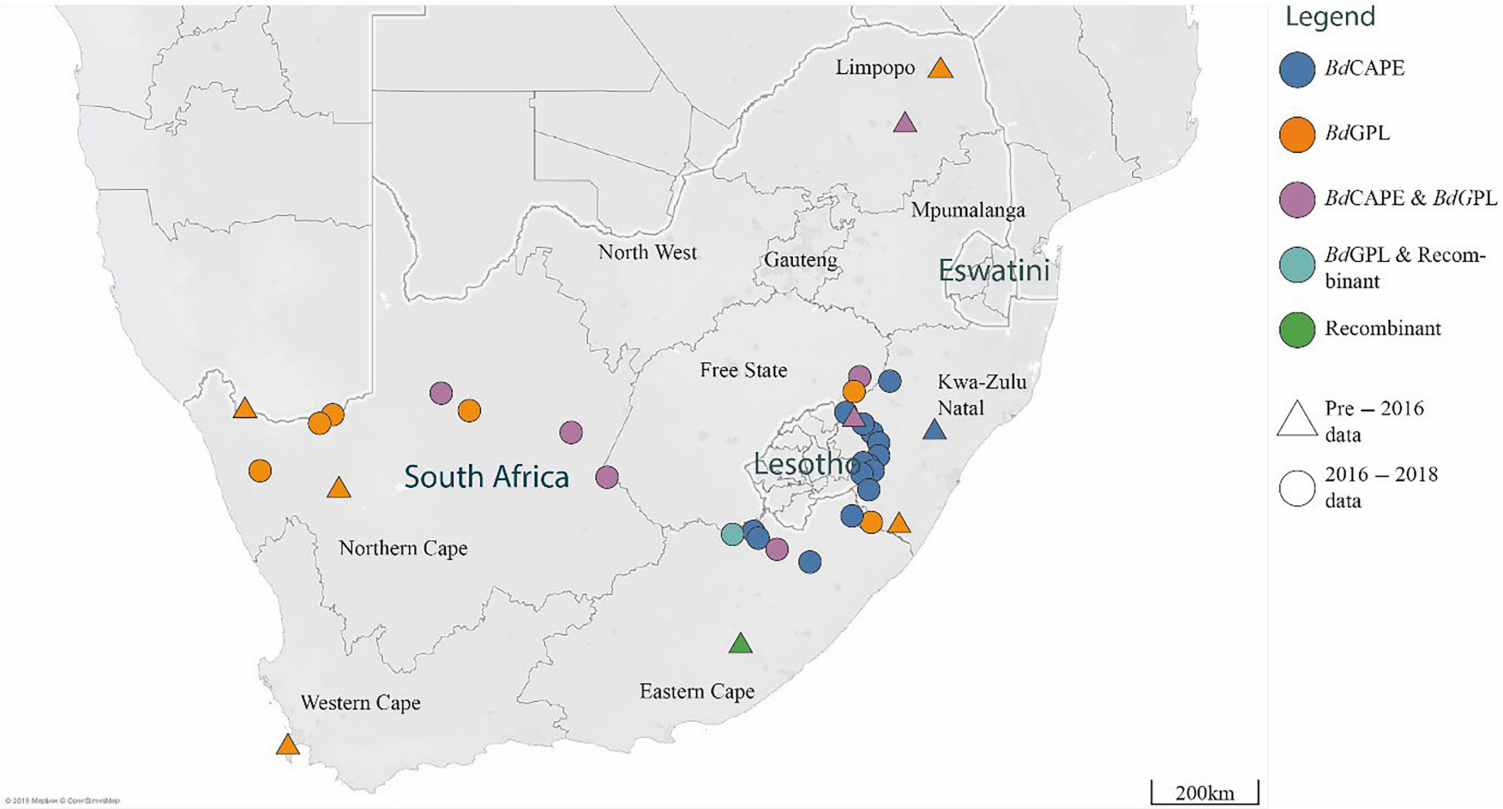
Distribution of *Bd* by lineage, indicating sites where both *Bd*GPL and *Bd*CAPE co‐occur (*Bd*CAPE and *Bd*GPL, as well as *Bd*GPL and Recombinant, signifies sites where both lineages were detected or isolated).

In total, we described six *Bd*GPL‐positive sites, 16 *Bd*CAPE‐positive sites, and six sites from which we collected either a recombinant isolate, recovered both lineages, or detected both lineages at a single site via qPCR lineage‐testing. A zone of regular lineage co‐occurrence was detected in the Northern Cape Province, another co‐occurrence zone was associated with the Drakensberg, and a third zone was detected in northern Limpopo (Figure 1).

### 3.2 Lineage association with climate

Single lineage sites differed in mean annual temperature, mean daily precipitation, and elevation (*p* < .05). Wilcoxon Rank Sum tests revealed significant differences in mean annual temperature and mean annual precipitation for *Bd*CAPE‐positive sites, compared to those that were positive for *Bd*GPL (*p* < .0001 and *p* = .0055, respectively) or both lineages (*p* = .0146 and *p* = .0002, respectively), but the same significant difference was not detected when comparing the mixed lineage sites to the *Bd*GPL‐positive sites (*p* = .2078 and *p* = .2365, respectively). Concerning elevation, *Bd*CAPE compared with *Bd*GPL, as well as *Bd*GPL compared with mixed lineage sites, differed significantly (*p* < .0001 and *p* = .0057, respectively). Mixed lineage sites were, however, not significantly different from *Bd*CAPE positive sites (*p* = .1815).

**FIGURE 2:**
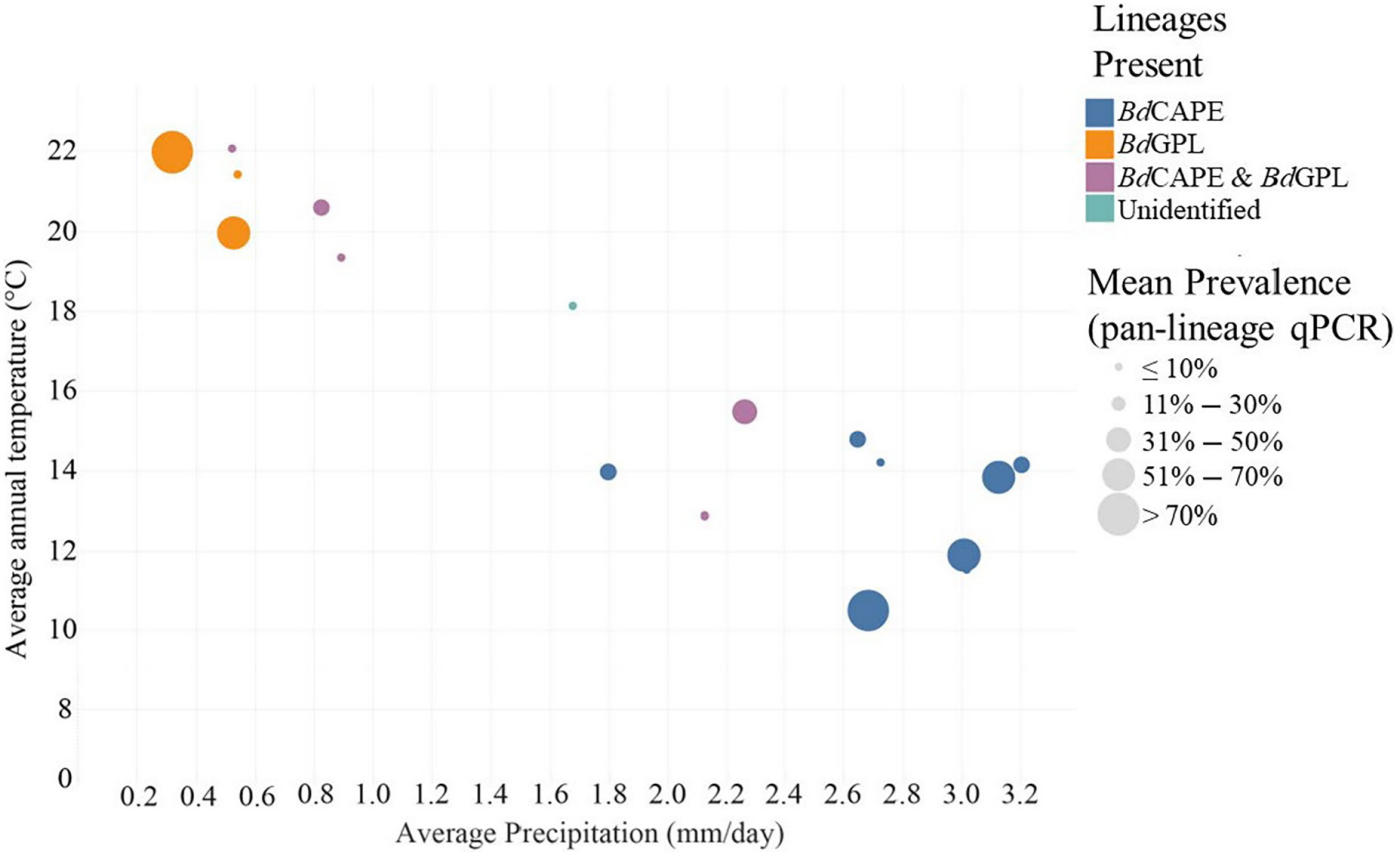
Scatterplot showing qPCR lineage‐typed sites according to average annual daily temperature and average annual daily precipitation.

Generally, *Bd*GPL was found in drier, warmer sites (Figure 2). The range of environmental parameters where *Bd*GPL occurred was wider than for *Bd*CAPE. Conversely, *Bd*CAPE occurrence was correlated with high precipitation, and low‐temperature environments. One exception was a *Bd*GPL isolate that originated from a more typical *Bd*CAPE area (Figure 1). When considering our data as visualized in Figure 2, it is apparent that the two basic lineages in South Africa have different core distributions separated by mean annual temperature and mean annual precipitation.

### 3.3 Lineage population structures

**FIGURE 3:**
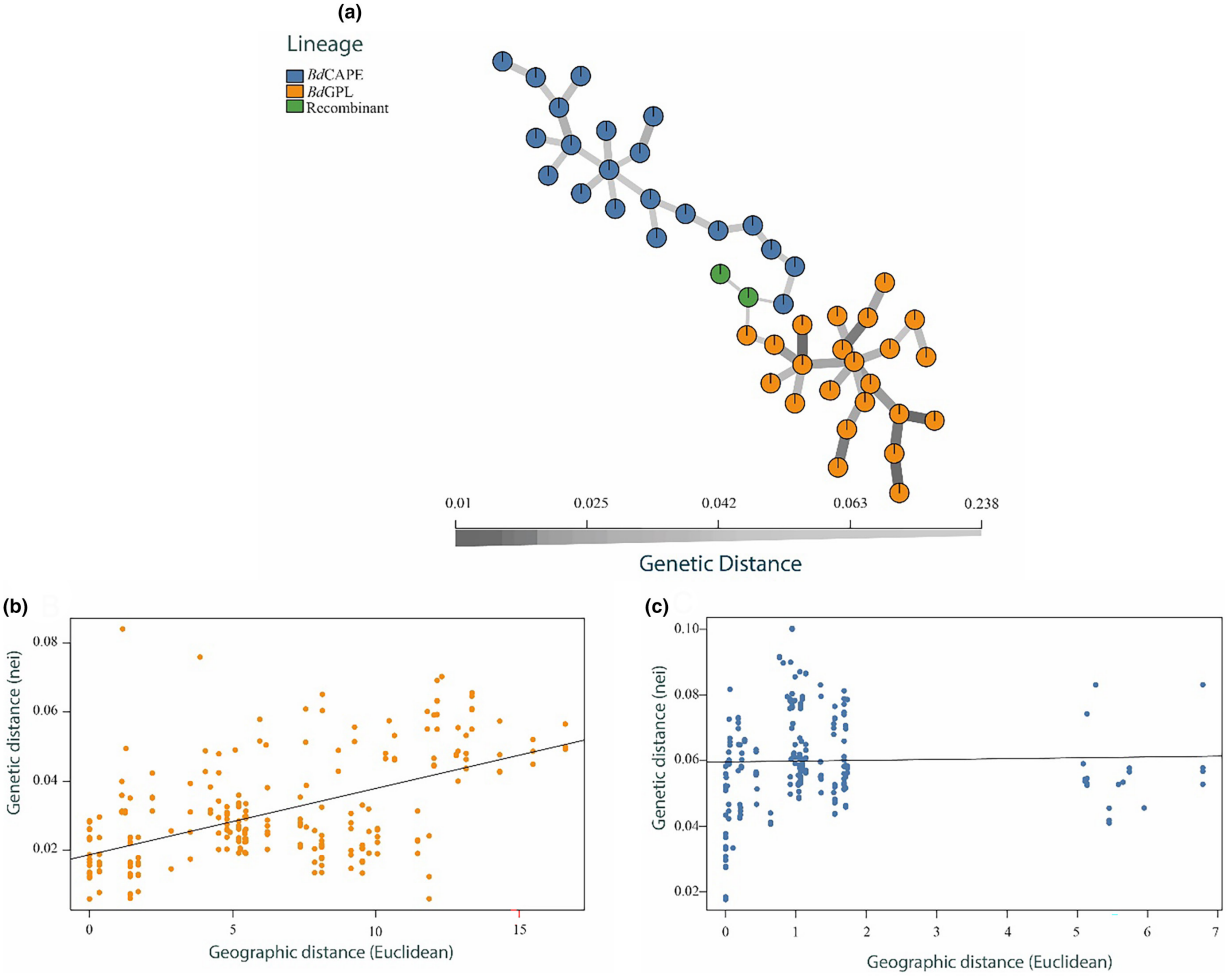
Comparative genomic analyses of *Bd* lineages (a) Minimum Spanning Network showing genetic distance between isolates. Closer genetic relatedness is illustrated by darker and thicker connecting lines between nodes, which represent individual isolates, (b) IBD plot for *Bd*GPL (*R* ^2^ = .31; *p* < .05), and (c) IBD plot for *Bd*CAPE (*R* ^2^ < .05; *p* > .05).

The MSN revealed greater clonality among *Bd*GPL isolates compared with *Bd*CAPE isolates (Figure 3a). The IBD analyses (Figure 3b, c) reveal that *Bd*GPL shows strong isolation by distance, with an *R* ^2^ value of .31 (*p* < .05). Conversely, *Bd*CAPE shows evidence of a more complex population structure with the isolates falling into two clusters and no significant correlation between genetic and geographic distance (*R* ^2^ < .05, *p* > .05). The two *Bd*CAPE genetic clusters are also temporally and geographically clustered, so further work will be required to resolve whether this is a compounding sampling effect or the results of a reproductive barrier.

### 3.4 Predicted *bd* lineage distribution

**FIGURE 4:**
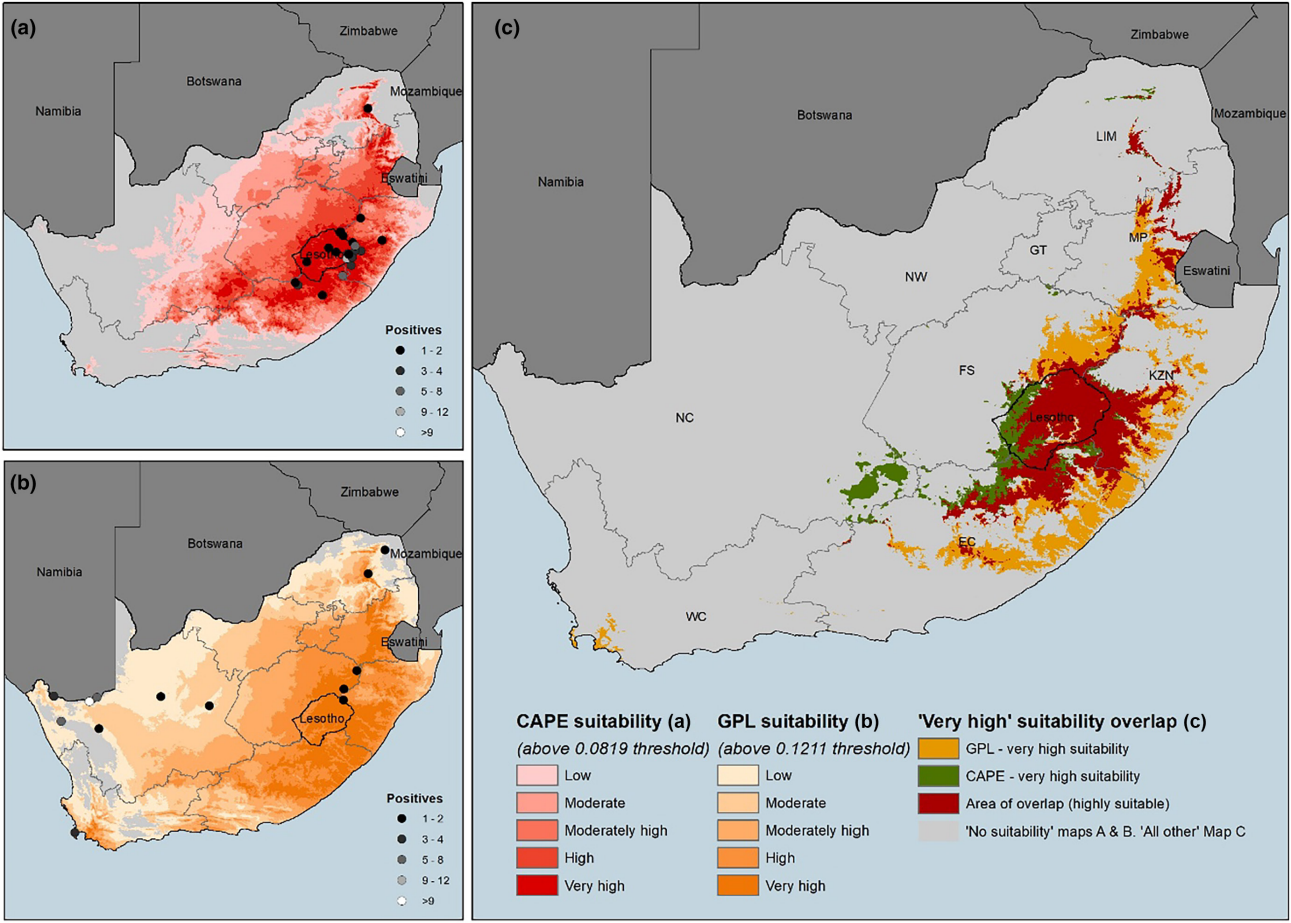
Suitability Maps for *Bd*CAPE (a) and *Bd*GPL (b). The “10th percentile training presence logistic threshold” is employed to differentiate between suitable and non‐suitable areas. Subsequently, suitable areas are divided into five categories using the Natural Breaks (Jenks) method. Map (c) illustrates the regions where both lineages have areas of very high suitability. South African Provinces: WC, Western Cape; NC, Northern Cape; EC, Eastern Cape; FS, Free State; NW, North West; GT, Gauteng; LIM, Limpopo; MP, Mpumalanga; KZN, Kwazulu‐Natal.

The database we constructed contained 1846 samples, originating from 48 species, 270 of which were positive records for *Bd*. Forty‐eight of the positive samples belonged to the *Bd*GPL, 128 samples to *Bd*CAPE, and 28 samples tested positive for both *Bd*GPL and *Bd*CAPE. Lineage had not been assigned for the remaining 66 positive samples. The lineage‐specific predicted distribution maps show that *Bd*CAPE has a restricted distribution, largely in eastern and southern coastal South Africa (Figure 4a). *Bd*GPL has an almost complete predicted distribution in South Africa including all areas where *Bd*CAPE is predicted to occur (Figure 4b). However, in the predicted hotspot for *Bd*CAPE, the eastern Great Escarpment, the environment is predicted to be highly suitable to *Bd*GPL (predicted above the threshold value of 0.1211—Figure 4b). Both models were strongly supported (*Bd*CAPE AUC: 0.968, *Bd*GPL AUC: 0.926). Analysis of the contribution of variables to the model showed that *Bd*GPL and *Bd*CAPE distributions were determined by different combinations of variables for both. The *Bd*CAPE model was influenced the most by the mean temperature of the driest quarter (at 54.2% contribution), while for *Bd*GPL, maximum temperature of the warmest month played the largest role (at 52.8%; Table 1). The resulting overlay of distribution indicated that the regions where both lineages include parts of Lesotho, the central and northern Drakensberg, the Eastern Cape, and south‐western KwaZulu‐Natal (Figure 4c).

**TABLE 1:** Bioclimatic variable contributions to the *Bd* lineage distribution models.

Key	Variable	Contribution to model	
		*Bd*GPL (%)	*Bd*CAPE (%)
Bio2	Mean Diurnal Range (Mean of monthly (max temp–min temp))	0.5	1.6
Bio3	Isothermality (BIO2/BIO7) (×100)	0.3	0.8
Bio5	Max Temperature of Warmest Month	52.8	15.9
Bio8	Mean Temperature of Wettest Quarter	0.6	1.1
Bio9	Mean Temperature of Driest Quarter	5.3	54.2
Bio11	Mean Temperature of Coldest Quarter	3.5	10.9
Bio13	Precipitation of Wettest Month	0.6	3.2
Bio14	Precipitation of Driest Month	5.1	0.8
Bio15	Precipitation Seasonality (Coefficient of Variation)	8.4	2.9
Bio19	Precipitation of Coldest Quarter	13.2	3.2
elev	Elevation above sea level	9.7	5.2

## 4 DISCUSSION

Out data showed that *Bd* lineages in South Africa are largely separated by environmental niche, with multiple co‐occurrence zones but only a single zone of overlap. *Bd*GPL was predominantly associated with low altitude environments that have a warmer, drier climate, and *Bd*CAPE mostly associated with cooler, higher precipitation environments. Both lineages show the potential for broader ecological niches as they sporadically occur within the opposing environmental envelope. It is within the contact zones that recombinant genotypes are expected to originate due to the higher frequency of mating opportunities between lineages.

For two fungal lineages to coexist, certain key factors are required such as sharing or partitioning of nutrients to reduce competition, spatial distribution (e.g., different lineages on separate hosts), or subtle differences in their physiological optimum that lead to seasonally driven temporal partitioning. Whatever facilitates coexistence, recombinant lineages may emerge because of sexual reproduction between lineages. These interactions among *Bd* lineages are rare and have only been described from surveillance and isolation efforts of *Bd* in South Africa and Brazil (Jenkinson et al., 2016; O'Hanlon et al., 2018; Schloegel et al., 2012). This stands in sharp contrast to the purported origin of amphibian associated chytrid fungi (AACs), Asia, where the greatest diversity of lineages has been identified but where no recombinants have been detected (O'Hanlon et al., 2018). *Bd* outside of Asia is an invasive pathogen, so areas of lineage overlap and recombination among lineages may be an outcome of anthropogenic globalization of AACs. Our data concur with previous studies showing that *Bd*GPL is less genetically diverse than *Bd*CAPE. The apparent presence of two clusters within the *Bd*CAPE population suggests at least two introduction events and perhaps some degree of reproductive barrier between sites of introduction, possibly due to altitudinal variation. However, the pattern may also be an artifact of sampling, which further surveillance could resolve. The observation of genetic isolation‐by‐distance for *Bd*GPL suggests that no reproductive barriers exist within their population, and points to what is likely a single introduction event; however, relatively low rates of gene flow with stepping‐stone dispersal from a single point of origin, unimpeded by geographical or environmental barriers, suggests reduced opportunity for intralineage recombination. This observation may also suggest that *Bd*GPL was more recently introduced into South Africa.

However, where contact zones are generated, recombination is a key process for generating genetic diversity in fungi, from which *Bd* is no exception. Most often recombination in fungi occurs during meiosis, but phenotypic or genetic characteristics associated with meiosis have yet to be described for *Bd*. Rather the presence of widespread chromosomal copy number variation suggests some form of parasexual recombination drives recombinant formation (Farrer et al., 2013; James et al., 2015). A likely outcome of genetic recombination regardless of the mechanism of reproduction is that it allows for adaptation to changing environments, especially since the recombinant we recovered was found in an ecotone area between the predominant sites of the parental lineages. The recombinant lineages may very well be better adapted to the ecotonal area than either of the parental lineages, but that may also mean that it may be less adapted to the home ranges of either or both parental lineages. Future experimental work to test virulence and transmission under different climatic conditions can provide further clarification.

Overall, genetic recombination in fungi requires the presence of compatible mating types, specific environmental conditions, physical proximity between individuals, and the recognition and fusion of gametes (Ni et al., 2011; Taylor et al., 1999). When conditions are favorable during sexual reproduction, the haploid nuclei of two different individuals must recognize and fuse with one another to form a diploid zygote. This requires the production of specialized structures, such as fruiting bodies or gametangia, that allow the gametes to come into contact and fuse. Although *Bd* is speculated to have similar structures, evidence of their existence has not been found and await discovery. Nevertheless, the recent discovery of recombinants in *Bd* validates that compatibility exists between multiple lineages but what constitutes the required environmental conditions for recombination in *Bd* has not been defined. Our data revealed ample evidence for lineages being in close proximity to one another, either at the same site within the same host population or on the same host. The region of recombination in South Africa emulates an ecotone where the area dominated by *Bd*CAPE transitions into a *Bd*GPL‐dominated area. Within this taxonomically defined geographical demarcation, both lineages are confronted by environmental change imposed by differences in climate. Within this area, the suitability of the environment means that the likelihood of individual lineages being brought into physical contact, through the actions of amphibians or through the inherent motility of zoospores, is increased. What the long‐term consequences of genetic recombinants entails for the conservation of wild amphibian populations have yet to be determined. However, an in vitro challenge experiment found that the *Bd*GPL and *Bd*ASIA‐2/BRAZIL recombinant was more virulent toward native hosts than either parental lineage (Greenspan et al., 2018).

Our landscape‐scale analysis of *Bd* lineages in South Africa underscores the need to concentrate future genetic epidemiology on contact zones where both lineages occur in order to further understand the consequence of recombination on epidemiologically relevant traits. More widely, there is a clear need to widen surveillance to areas predicted to be suitable for the establishment of *Bd* in order to assess the risk that this pathogen poses to amphibians in Africa.

## AUTHOR CONTRIBUTIONS

**Ruhan Verster:** Conceptualization (equal); investigation (equal); methodology (equal); project administration (equal); validation (supporting); visualization (supporting); writing – original draft (lead); writing – review and editing (equal). **Pria Natalia Ghosh:** Conceptualization (equal); data curation (equal); formal analysis (equal); visualization (equal); writing – review and editing (equal). **Tom R Sewell:** Conceptualization (equal); formal analysis (equal); investigation (equal); methodology (equal); visualization (equal); writing – review and editing (equal). **Trenton W. J. Garner:** Conceptualization (equal); funding acquisition (equal); writing – review and editing (equal). **Matthew Fisher:** Conceptualization (equal); funding acquisition (equal); writing – review and editing (equal). **Wynand Muller:** Visualization (equal); writing – review and editing (equal). **Dirk Cilliers:** Visualization (equal); writing – review and editing (equal). **Weldon Che:** Conceptualization (equal); project administration (lead); resources (equal); supervision (lead); writing – review and editing (lead).

## Supporting information


Data S1.

**Figure S1.** **–S3.**
Table S1.–S3.

## Data Availability

The dataset used for the predictive model was obtained from publications and our fieldwork and is available from the DRYAD database link in the Appendix. All the authors have no conflict of interest to declare.
