# Supplementary material for: Environment predicts Batrachochytrium dendrobatidis lineage distribution and zones of recombination in South Africa
Source: Ecol Evol. 2024 Feb 22;14(2):e11037. doi: 10.1002/ece3.11037 (PMC10883246; doi:10.1002/ece3.11037)
Supplement: Supplementary file 2 — Figure S1. –S3. Table S1.–S3. [file ECE3-14-e11037-s002.docx]

# **Appendix**

Jack-knife test results for the predicted distribution models pertaining to Figures 4(A-C). Note that without variable indicates the effect removing the specific parameter will have on the models, with only variable indicates the performance of the model using the specific parameter in isolation and with all variables indicate the AUC score if all variables are used.


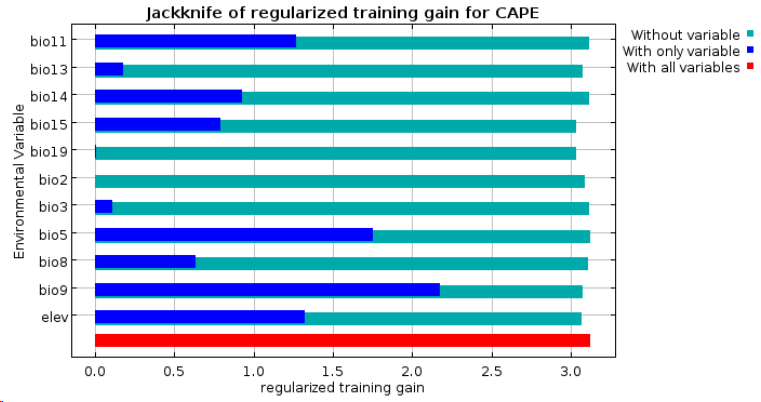


Figure S1: Jack-knife analyses of the environmental variables contributing to the final prediction model of *Bd*CAPE in South Africa


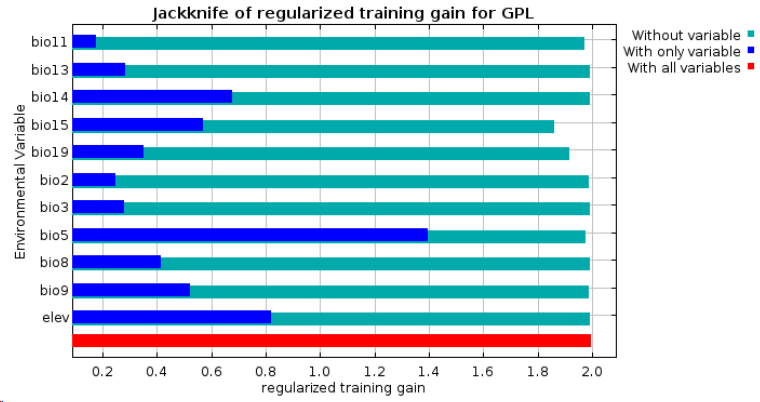


Figure S2: Jack-knife analyses of the environmental variables contributing to the final prediction model of *Bd*GPL in South Africa


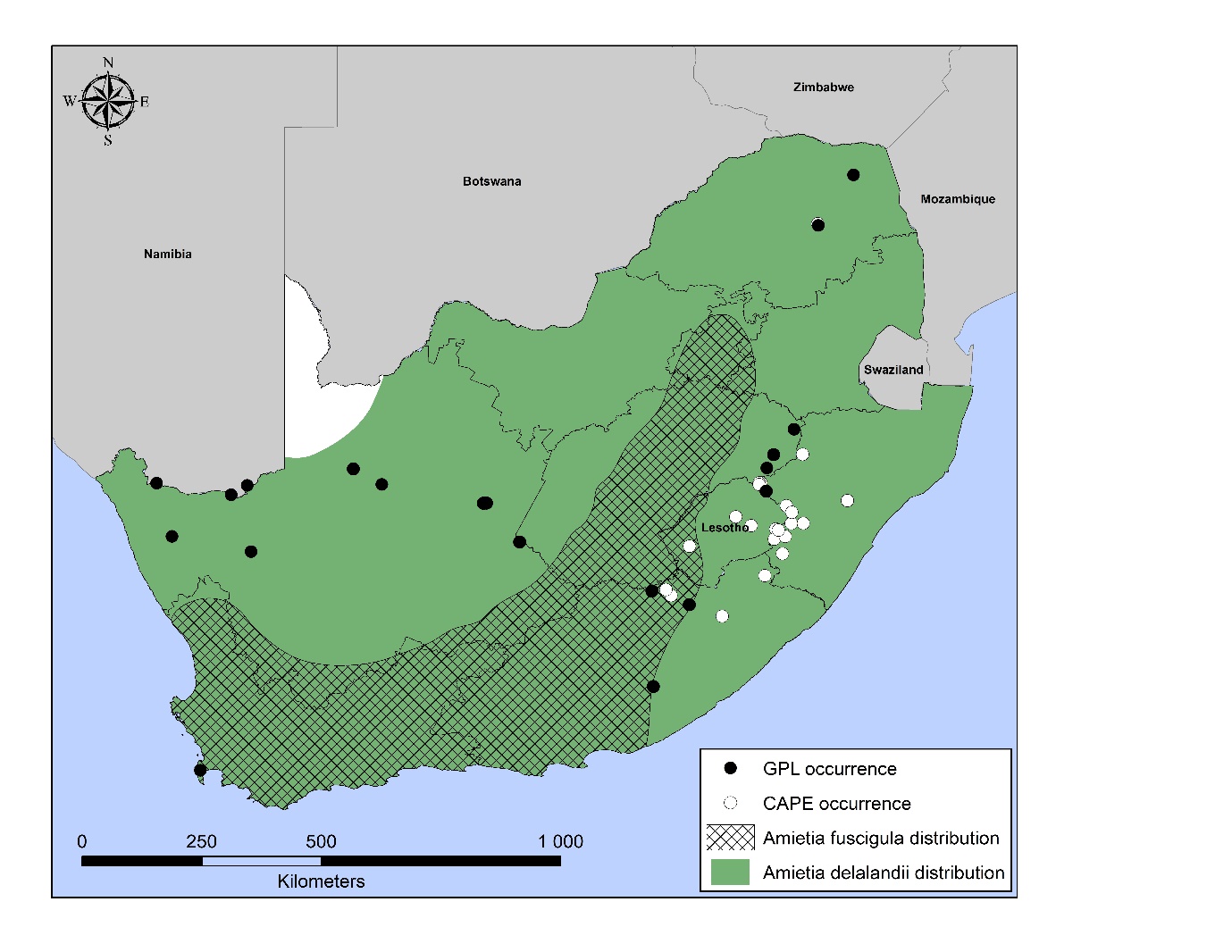
Figure S3: Predominant *Bd* lineage at study sites, overlaid on the distribution of *Amietia delalandii* and *A. fuscigula* within South Africa.

Table S1: Summary of different species were sampled, with date and proportion to total database contribution in percentage

| **Species** | **Year sampled** | **No. (positives)** | **Proportion of contribution**  **(% positives)** | **Lineage found (*)** |
| --- | --- | --- | --- | --- |
| *Afrixalus brachycnemis* | 2002 | 4 (0) | 0.22 (0) |  |
| *Afrixalus crotalus* | 2002 | 4 (1) | 0.22 (0.37) | n/a |
| *Afrixalus fornasini* | 2017 | 1 (0) | 0.05 (0) |  |
| *Amietia angolensis* | 2002 | 16 (4) | 0.87 (1.48) | n/a |
| *Amietia delalandi* | 2008, 2010, 2015-2018 | 696 (99) | 37.70 (36.67) | *Bd*CAPE and *Bd*GPL |
| *Amietia dracomontana* | 2004 | 6 (5) | 0.33 (1.85) | *Bd*CAPE |
| *Amietia fuscigula* | 2008, 2016, 2018 | 184 (43) | 9.97 (15.93) | *Bd*CAPE and *Bd*GPL |
| *Amietia hymenopus* | 2016 | 53 (24) | 2.87 (8.89) | *Bd*CAPE |
| *Amietia johnstoni* | 2002 | 7 (2) | 0.38 (0.74) | n/a |
| *Amietia poyntoni* | 2017-2018 | 34 (6) | 1.84 (2.22) | *Bd*CAPE |
| *Amietia vandijki* | 2002 | 2 (0) | 0.11 (0) |  |
| *Amietia vertebralis* | 2000, 2004, 2008, 2018 | 30 (9) | 1.63 (3.33) | *Bd*CAPE |
| *Amietia* sp. | 2017 | 16 (3) | 0.87 (0.110) | *Bd*CAPE and *Bd*GPL |
| *Arthroleptis francei* | 2002 | 1 (0) | 0.05 (0) | n/a |
| *Arthroleptis stenodactylus* | 2002 | 1 (0) | 0.05 (0) | n/a |
| *Arthroleptis xenodactyloides* | 2002 | 3 (2) | 0.16 (0.74) | n/a |
| *Arthroleptis* sp. 1 | 2002 | 1 (0) | 0.05 (0) | n/a |
| *Arthroleptis* sp. 2 | 2002 | 1 (0) | 0.05 (0) | n/a |
| *Cacosternum boettgeri* | 2016 | 1 (0) | 0.05 (0) |  |
| *Cacosternum nanum* | 2017 | 41 (0) | 2.22 (0) |  |
| *Cacosternum* spp. | 2016 | 35 (0) | 1.90 (0) |  |
| *Capensibufo rosei* | 2002 | 1 (0) | 0.05 (0) |  |
| *Chiromantis xerampelina* | 2017-2018 | 14 (0) | 0.76 (0) |  |
| *Hadromophryne natalensis* | 2008, 2010, 2016-2017 | 57 (34) | 3.09 (12.59) | *Bd*CAPE |
| *Hemisus guttatus* | 2017 | 1 (0) | 0.05 (0) |  |
| *Hemisus marmoratus* | 2002 | 2 (0) | 0.11 (0) |  |
| *Hyperolius tuberilinguis* | 2017 | 4 (0) | 0.22 (0) |  |
| *Kassina senegalensis* | 2017-2018 | 89 (0) | 4.82 (0) |  |
| *assin asp*. | 2016 | 25 (0) | 1.35 (0) |  |
| *Leptopelis argenteus* | 2002 | 4 (0) | 0.22 (0) |  |
| *Leptopelis flavomaculatus* | 2002 | 1 (0) | 0.05 (0) |  |
| *Leptopelis mossambicus* | 2017 | 3 (0) | 0.16 (0) |  |
| *Nothophryne broadleyi* | 2002 | 4 (1) | 0.22 (0.37) | n/a |
| *Phrynobatrachus natalensis* | 2002 | 4 (2) | 0.22 (0.74) | n/a |
| *Ptychadena anchietae* | 2002 | 3 (0) | 0.16 (0) |  |
| *Ptychadena porosissima* | 2017 | 1 (0) | 0.05 (0) |  |
| *Pyxichephalus edulis* | 2017 | 1 (0) | 0.05 (0) |  |
| *Schismaderma carens* | 2018 | 27 (0) | 1.46 (0) |  |
| *Sclerophrys capensis* | 2017 | 51 (0) | 2.76 (0) |  |
| *Sclerophrys gutturalis* | 2016-2017 | 56 (0) | 3.03 (0) |  |
| *Sclerophrys maculata* | 2002 | 2 (0) | 0.11 (0) |  |
| *Sclerophrys poweri* | 2016-2017 | 8 (0) | 0.43 (0) |  |
| *Semnodactylus weallii* | 2018 | 28 (1) | 1.52 (0.37) | *Bd*CAPE |
| *Strongylopus bonaespei* | 2002 | 1 (0) | 0.05 (0) |  |
| *Strongylopus fasciatus* | 2018 | 1 (0) | 0.05 (0) |  |
| *Strongylopus fuelleborni* | 2002 | 2 (1) | 0.11 (0.37) | n/a |
| *Strongylopus grayii* | 2002, 2016-2018 | 26 (1) | 1.41 (0.37) | n/a |
| *Tomopterna cryptotis* | 2016-2017 | 17 (1) | 0.92 (0.37) | *Bd*GPL |
| *Tomopterna natalensis* | 2017 | 1 (0) | 0.05 (0) |  |
| *Tomopterna* sp. | 2017 | 2 (0) | 0.11 (0) |  |
| *Vandijkophrynus gariepensis* | 2016 | 16 (8) | 0.87 (2.96) | *Bd*GPL |
| *Xenopus laevis* | 2005, 2008, 2016-2017 | 14 (2) | 0.76 (0.74) | *Bd*GPL |
| *Xenopus muelleri* | 1991, 1998 | 8 (3) | 0.43 (1.11) | n/a |
| Species unavailable | Not Available | 235 (18) | 12.73 (6.67) |  |
| **Total** | | **1846 (270)** |  |  |

(*) – n/a refers to samples that tested positive, but the lineage identification is not known, either due to isolation of cultures being impossible or swab DNA for lineage-specific qPCR’s being unavailable

|  | **fc** | **rm** | **auc.val.avg** | **auc.val.sd** | **delta.AICc** |
| --- | --- | --- | --- | --- | --- |
| 1 | L | 0.5 | 0.952961111 | 0.668237998 | 28.62319059 |
| 2 | LQ | 0.5 | 0.972138889 | 0.527881818 | 0 |
| 3 | H | 0.5 | 0.975608333 | 0.468118728 | NA |
| 4 | LQH | 0.5 | 0.973780556 | 0.562801876 | 1108.815023 |
| 5 | LQHP | 0.5 | 0.972569444 | 0.623302998 | 1105.375724 |
| 6 | LQHPT | 0.5 | 0.973508333 | 0.564932709 | NA |
| 7 | L | 1.5 | 0.953922222 | 0.655192639 | 29.07240324 |
| 8 | LQ | 1.5 | 0.96675 | 0.536122106 | 23.29633023 |
| 9 | H | 1.5 | 0.973455556 | 0.457524544 | 61.6711714 |
| 10 | LQH | 1.5 | 0.972530556 | 0.530267901 | 49.22335935 |
| 11 | LQHP | 1.5 | 0.972386111 | 0.538918196 | 36.53331517 |
| 12 | LQHPT | 1.5 | 0.971738889 | 0.540813279 | 26.30787163 |
| 13 | L | 2.5 | 0.953825 | 0.645018765 | 29.97834877 |
| 14 | LQ | 2.5 | 0.960261111 | 0.569140546 | 29.36255136 |
| 15 | H | 2.5 | 0.971363889 | 0.420552487 | 13.04761364 |
| 16 | LQH | 2.5 | 0.968747222 | 0.523252229 | 37.16780717 |
| 17 | LQHP | 2.5 | 0.970255556 | 0.498663403 | 6.099722907 |
| 18 | LQHPT | 2.5 | 0.969886111 | 0.500971498 | 6.099722907 |
| 19 | L | 3.5 | 0.95195 | 0.664756937 | 34.96681532 |
| 20 | LQ | 3.5 | 0.955652778 | 0.609917723 | 30.48756917 |
| 21 | H | 3.5 | 0.971102778 | 0.367688007 | 23.4768034 |
| 22 | LQH | 3.5 | 0.968258333 | 0.488197326 | 17.07412607 |
| 23 | LQHP | 3.5 | 0.969258333 | 0.471238942 | 13.29377028 |
| 24 | LQHPT | 3.5 | 0.969258333 | 0.471238942 | 13.29377028 |
| 25 | L | 4.5 | 0.949605556 | 0.691359098 | 40.63928794 |
| 26 | LQ | 4.5 | 0.953202778 | 0.64072556 | 29.11834429 |
| 27 | H | 4.5 | 0.971666667 | 0.331106084 | 23.66789879 |
| 28 | LQH | 4.5 | 0.968613889 | 0.454207219 | 12.37382448 |
| 29 | LQHP | 4.5 | 0.968761111 | 0.469600161 | 14.06502269 |
| 30 | LQHPT | 4.5 | 0.968761111 | 0.469600161 | 14.06502269 |

Table S2: AUC and AICc values for the *Bd*CAPE model

|  | **fc** | **rm** | **auc.val.avg** | **auc.val.sd** | **delta.AICc** |
| --- | --- | --- | --- | --- | --- |
| 1 | L | 0.5 | 0.905531148 | 1.036471674 | 30.6471437 |
| 2 | LQ | 0.5 | 0.921965574 | 0.852912452 | 0 |
| 3 | H | 0.5 | 0.936540984 | 0.685551854 | 158.4955382 |
| 4 | LQH | 0.5 | 0.932091803 | 0.723835689 | 340.0855771 |
| 5 | LQHP | 0.5 | 0.946545902 | 0.60793872 | 243.730076 |
| 6 | LQHPT | 0.5 | 0.94024918 | 0.669729605 | NA |
| 7 | L | 1.5 | 0.904632787 | 1.0812067 | 32.06630105 |
| 8 | LQ | 1.5 | 0.918995082 | 0.89772479 | 11.18887321 |
| 9 | H | 1.5 | 0.935277049 | 0.671110981 | 12.05592836 |
| 10 | LQH | 1.5 | 0.92342459 | 0.832661724 | 9.241927714 |
| 11 | LQHP | 1.5 | 0.93034918 | 0.778799361 | 8.720530605 |
| 12 | LQHPT | 1.5 | 0.927296721 | 0.781966625 | 5.580560605 |
| 13 | L | 2.5 | 0.903940984 | 1.110235149 | 31.29866882 |
| 14 | LQ | 2.5 | 0.91622459 | 0.947959842 | 16.85074734 |
| 15 | H | 2.5 | 0.92197377 | 0.840015186 | 27.33710782 |
| 16 | LQH | 2.5 | 0.918442623 | 0.925828077 | 9.218408011 |
| 17 | LQHP | 2.5 | 0.921701639 | 0.91729428 | 3.139207697 |
| 18 | LQHPT | 2.5 | 0.91834918 | 0.930621565 | 6.391160698 |
| 19 | L | 3.5 | 0.903319672 | 1.125561034 | 34.03529208 |
| 20 | LQ | 3.5 | 0.913919672 | 0.984056604 | 21.85948168 |
| 21 | H | 3.5 | 0.907711475 | 1.033494258 | 21.88631819 |
| 22 | LQH | 3.5 | 0.916378689 | 0.951984262 | 24.08409022 |
| 23 | LQHP | 3.5 | 0.913190164 | 0.997577308 | 27.74924727 |
| 24 | LQHPT | 3.5 | 0.910921311 | 1.004062755 | 27.74924727 |
| 25 | L | 4.5 | 0.902672131 | 1.138543324 | 34.15962767 |
| 26 | LQ | 4.5 | 0.912098361 | 1.001031902 | 25.75019418 |
| 27 | H | 4.5 | 0.89924918 | 1.130333579 | 30.90327787 |
| 28 | LQH | 4.5 | 0.913232787 | 0.986586442 | 18.98684161 |
| 29 | LQHP | 4.5 | 0.909568852 | 1.041678619 | 23.91302166 |
| 30 | LQHPT | 4.5 | 0.908414754 | 1.043476756 | 23.91302166 |

Table S3: AUC and AICc values for the *Bd*GPL model

Dataset for Predictive models obtainable at: <https://datadryad.org/stash/share/BlPmJ1-QNkWn95RK465VWKet1pLqVxJ3fUVdT13CbBw>
